# STING Agonist Combined to a Protein-Based Cancer Vaccine Potentiates Peripheral and Intra-Tumoral T Cell Immunity

**DOI:** 10.3389/fimmu.2021.695056

**Published:** 2021-07-01

**Authors:** Matteo Rossi, Susanna Carboni, Wilma Di Berardino-Besson, Erika Riva, Marie-Laure Santiago-Raber, Elodie Belnoue, Madiha Derouazi

**Affiliations:** ^1^ AMAL Therapeutics, Geneva, Switzerland; ^2^ Boehringer Ingelheim International GmbH, Ingelheim, Germany

**Keywords:** STING agonist, protein cancer vaccine, combination immunotherapy, CD8 T cells functionality, Th1 CD4 T cells, tumor microenvironment

## Abstract

Combining different immunotherapy approaches is currently building the future of immunotherapy, with the view to maximize anti-tumoral efficacy for larger patient population. The KISIMA™ platform allows the development of protein-based cancer vaccines able to induce tumor-specific T cell response resulting in anti-tumoral efficacy in various mouse models. Intra-tumoral administration of stimulator of interferon gene agonists (STINGa) was shown to induce a potent inflammatory response leading to the development of tumor-specific immunity. Here, we explored the efficacy and mechanisms of action of subcutaneous STINGa treatment combined with therapeutic vaccination in various mouse tumor models. This combinatory treatment highly enhanced frequency and effector function of both peripheral and intra-tumoral antigen-specific CD8 T cells, promoting potent IFN*γ* and TNFα production along with increased cytotoxicity. Moreover, combination therapy favorably modulated the tumor microenvironment by dampening immune-suppressive cells and increasing CD4 T cell infiltration together with their polarization toward Th1 phenotype. Combination with STINGa treatment improved the effect of therapeutic vaccination, resulting in a prolonged control and slower growth of B16-OVA and TC-1 tumors. Altogether, the results presented here highlight the potential of combining STINGa with a therapeutic protein vaccine for cancer treatment.

## Introduction

It is now established that modulating the immune system of cancer patients to specifically recognize and eliminate tumor cells is a promising therapeutic modality. As of September 2020, 4,400 clinical trials investigating the efficacy of PD-1/PD-L1 blockade were open (1). The initial enthusiasm for the impressive efficacy of checkpoint inhibitors (CPIs) was nevertheless dampened by the restricted patient population responding to therapy and by acquired treatment resistance (2).

In parallel to immune checkpoints, immune co-stimulators—molecules transiently expressed or up-regulated by T cells during the priming to potently increase their activation such as OX40 or Glucocorticoid-induced TNFR-related protein (GITR)—are promising immunotherapy targets. OX40- and GITR-agonists have been shown to potently stimulate anti-tumoral immune response in pre-clinical studies, resulting in an inhibition of tumor growth (3, 4). Another very promising strategy is the targeting of the stimulator of interferon genes (STING) pathway. STING is an adaptor protein activated by the binding to cyclic GAMP—a by-product of cytosolic DNA degradation by DNA sensors (5, 6)—which upon activation induces the secretion of high levels of type I interferons and other pro-inflammatory cytokines such as IL-6 and TNFα (7–9). Activation of STING was shown to enhance NK cell recruitment and activation (10) and to promote CD4 and CD8 T cell chemotaxis (11). In addition, STING signaling was found to be inhibited in patient derived colorectal adenocarcinoma cells, supporting its anti-tumoral role (12). Due to these properties, synthetic STING agonists have been tested in pre-clinical and clinical studies with the intent of inflaming the tumor and eliciting an anti-tumoral immune response. Intra-tumoral injection of STING agonist was shown to induce tumor regression as well as a systemic, tumor-specific memory immune response in different murine tumor models (13). Moreover, STING agonist formulated within a GM-CSF-producing cancer cell vaccine was shown to delay tumor progression in different murine models, demonstrating that intra-tumoral administration is not the only effective route (14). Currently, multiple phase 1/2 clinical trials investigate the use of STING agonists—alone or in combination with CPIs—in different solid tumors and lymphoma patients (15).

As for CPIs, the impact of immune-modulators is however limited by the need for a pre-existing immune response able to infiltrate the core of the tumor, while cancer cells are master of immune evasion and suppression. Combination of immune modulators with therapeutic cancer vaccines could support T cell-mediated immunity and infiltration in the tumor despite a detrimental suppressive tumor micro-environment (16, 17).

We previously described an original chimeric protein vaccine platform, named KISIMA™, composed of three elements: a ZEBRA-derived cell-penetrating peptide (Z13) (18), a multiantigenic domain (Mad) with epitopes restricted by multiple MHC alleles, and a TLR2/4 agonist (Anaxa) conferring self-adjuvanticity. This vaccine platform was shown to elicit both CD8 and CD4 antigen-specific T cell responses in preclinical tumor models, leading to immunological memory and high vaccine efficacy (19, 20). Here, we sought to take advantage of both cancer vaccine and immune-modulator properties to impact not only the quantity but also the quality of both CD8 and CD4 T cells, establishing a combination immunotherapy able to effectively tackle different types of cancers. We assessed the combination of KISIMA-derived vaccines with subcutaneously administered STING agonist. Improved tumor growth control in mouse tumor models was associated with higher frequency of CD8 and CD4 T cells, improved effector functions, re-polarization of CD4 toward Th1, and modulation of the tumor microenvironment (TME).

## Materials And Methods

### Mice

Female C57BL/6J mice were purchased from Charles River Laboratories (L’arbresle, France). All animals used in this study were between 6 and 10 weeks old at the time of experiments.

### Vaccines

Vaccine constructs were designed in-house and produced in *E. coli* by Genscript. Vaccines were prepared by dilution in vaccine buffer (50 mM TRIS-HCl, 150 mM NaCl, 10% Glycerol, 1 M L-Arg, 1mM DTT, 0.2% Tween20, pH 8) and administered by subcutaneous (s.c.) injection of 10 nmoles in 100 μl volume. The different constructions used are illustrated in **Figure S1**.

### STING Agonist

STING agonist (ML-RR-S2 CDA, ADU-S100, Med Chem Express) was resuspended in DMSO at a concentration of 6.9 mg/ml and diluted in 1× phosphate buffer saline (PBS, Gibco) prior to injection.

### Tumor-Free Mice Vaccination Experiments

C57BL/6 mice were vaccinated twice (at days 0 and 14 for Z13Mad25Anaxa and days 0 and 7 for Z13Mad39Anaxa) by s.c. injection of 10 nmoles of vaccine at the tail base. At the same time of vaccination, mice received 25 μg of STING agonist administered *via* 2× 50 μl s.c. injections in each side of the low back, in proximity of the vaccination site. Serum was collected 4 and 24 h after the first vaccination and IFN-α concentration was measured by ELISA. Whole blood was collected one week after the last vaccination and used for antigen-specific CD8 T cell measurement by multimer flow cytometry staining. At the same time, spleens were harvested, and splenocytes were used for *ex vivo* stimulation, and intracellular cytokine production was analyzed by flow cytometry. Alternatively, splenocytes were used for TCR avidity assay.

### Tumor Cell Line

The TC-1 cell line was obtained from ATCC. This cell line, derived from lung epithelial cells transfected with HPV16 E6/E7 and c-H-ras oncogenes, was maintained in RPMI 1640 Glutamax™ supplemented with 10% heat-inactivated fetal calf serum (FCS), 100 U/ml penicillin/streptomycin (P/S), 1 mM sodium pyruvate, MEM NEAA, and 0.4 mg/ml geneticin G418.

The B16-OVA cell line was provided by Bertrand Huard (University of Grenoble-Alpes, France). This cell line, derived from mouse melanoma cells transfected with OVA, was maintained in RPMI 1640 Glutamax™ supplemented with 10% heat-inactivated fetal calf serum (FCS), 100 U/ml penicillin/streptomycin (P/S), 1 mM sodium pyruvate, MEM NEAA and 1 mg/ml geneticin G418.

### *In Vivo *Tumor Experiments

C57BL/6J mice were implanted s.c. with 1 × 10^5^ TC-1 tumor cells in the back, and mice were stratified according to tumor size on day 6 of tumor implantation. Alternatively, C57BL/6J mice were injected i.v. with 1 × 10^5^ B16-OVA cells. Mice were vaccinated two times by s.c. injection of 10 nmoles of KISIMA vaccine at the tail base. At the same time of vaccination, mice received 25 μg of STING agonist administered *via* 2× 50 μl s.c. injections in each side of the low back, in proximity of the vaccination site. TC-1 tumor size was measured with a caliper, and mice were euthanized when tumor reached a volume of 1,000 mm^3^. Tumor volume was calculated with the following formula: *V = length* × *length* × *width* × *Pi/6*. B16-OVA tumor bearing mice were sacrificed at day 20; lungs were perfused with a saline solution, and the number of lung metastasis was counted.

### *Ex Vivo *Cell Preparation

TC-1 tumors were harvested at day 20 post implantation, and tumor-infiltrating leucocytes (TILs) were purified using mouse tumor dissociation kit from Miltenyi, following manufacturer’s instructions. Briefly, tumor tissues were chopped into small pieces and resuspended in DMEM medium containing tumor dissociating enzymes (Miltenyi). Tumors were digested on a Gentle MACS with heating system (Miltenyi) using solid tumor program. Enzymatic digestion was stopped by adding cold PBS 0.5% BSA solution and keeping cells on ice. Digested tumors were passed through a 70 μm cell strainer to eliminate remaining undigested tissue. CD45+ cells were purified using CD45 TIL microbeads (Miltenyi) following manufacturer’s protocol. Purified CD45+ cells were used for flow cytometry staining or *ex vivo* T cell stimulation.

B16-OVA tumor bearing mice were perfused with a saline solution to eliminate blood from the lungs before their collection. Lung-infiltrating leucocytes (LILs) were purified using mouse tumor dissociation kit from Miltenyi, following manufacturer’s instructions.

Peripheral blood and spleen mononuclear cell suspensions from mice were isolated using Ficoll–Paque gradient (GE Healthcare) before flow cytometry analysis, *ex vivo* stimulation, or TCR avidity assay.

### *Ex Vivo *T Cell Stimulation

TILs, LILs, or splenocytes were numerated, and 1 × 10^5^ or 2 × 10^6^ cells were plated per condition, respectively. Cells were incubated with HPV-CD8, OVA-CD8, or OVA-CD4 epitope peptide, or without any stimulant as a negative control in the presence of Golgi stop (BD biosciences) and a fluorochrome coupled anti-CD107α for 6 h. After washing, cells were stained for cell surface antigens and fixable viability dye, then, after fixation and permeabilization according to manufacturer’s instructions (BD biosciences), cells were stained for intracellular cytokines.

### *In Vivo *Cytotoxicity Assay

Naive splenocytes were harvested and incubated for 1.5 h in DMEM complete medium at 37°C with or without HPV-E7 CD8 epitope peptide. Then, loaded and non-loaded splenocytes were stained with cell tracer violet (CTV) or CFSE (both from ThermoFisher Scientific), respectively, following manufacturer’s instructions. Splenocytes were then mixed at a 1:1 ratio, and a total of 5 × 10^6^ cells were transferred by intravenous injection into previously vaccinated mice. Then 20 h post cell transfer, splenocytes were harvested, and the survival of CTV or CFSE stained cells was assessed by flow cytometry. The percentage of antigen-specific killing was calculated with the following formula: % antigen-specific killing = (1−(ratio peptide^+^: peptide^-^ vaccinated/ratio peptide^+^: peptide^−^ naive)) * 100.

### *Ex Vivo *TCR Avidity Assay

One week after the second vaccination, spleens were harvested, and splenocytes were isolated (see above). Then 1 × 10^6^ cells/well were seeded in an IFN-*γ* ELISpot plate (Diaclone) and stimulated overnight with decreasing concentrations of RAHYNIVTF or SIINFEKL peptide. ELISpot plates were then revealed following manufacturer’s instructions, and the percentage of maximal response was calculated relatively to the highest concentration of stimulating peptide.

### Antibodies and Flow Cytometry

The following antibodies were used: CD45 (clone 30-F11), CD11b (M1/70), KLRG1 (2F1), CD103 (M290), NKg2a (20d5), Ly6C (AL-21), Ly6G (1A8), PD-L1 (MIH5), I-A/I-E (M5/114), CD11c (HL3), PDCA1 (927), CD64 (X54-5/7.1), B220 (RA3-6B2), CD24 (M1/69), CD4 (GK1.5), CD25 (3C7), CD3 (500A2), NKp46 (29A1.4), TNF-α (MP6-XT22), IFN-γ (XMG1.2), H2-Kb (AF6-88.5), and H2-Db (28–14–8) were from BD Biosciences; Tim3 (RMT3-23), PD-1 (29F.1A12), CD38 (90), Gr-1 (RB6-8C5), CD206 (C068C2), CD68 (FA-11) were from BioLegend; FoxP3 (FJK-16s), T-bet (4B10), GATA-3 (TWAJ), and ROR*γ*t (AFKJS-9) were from ThermoFisher Scientific; Granzyme B (REA226) was from Miltenyi; CD8 (KT15) was from MBL. Dead cells were stained with LIVE/DEAD Yellow or Aqua fluorescent reactive dye (Life Technologies) and excluded from analyses. Murine MHC-peptide multimers were from Immudex (Copenhagen, Denmark). Cells were analyzed using an Attune NxT flow cytometer (ThermoFisher Scientific), and results were analyzed with Kaluza (Beckman Coulter) software.

### Quantification of Serum Interferon*-α*

Blood was collected from mouse tail vein, and serum was isolated by centrifugation using Starstedt tubes. The concentration of IFN-α cytokine was measured using commercial ELISA kits according to the manufacturer’s recommendations (PBL Assay Science).

### HPV-16 E7 mRNA Extraction and Sequencing

A tumor sample of 4 mm^2^ was snap dry frozen in liquid nitrogen and RNA was extracted using the RNwasy Plus Mini kit (Qiagen) following manufacturer’s instructions. cDNA was generated by RT-PCR, and HPV-16 E7 DNA was then amplified using the following primers: Forward 5′-ATGCATGGAGATACACCTAC-3′; Reverse 5′-TTATGGTTTCTGAGAACAGATG-3′. The amplified cDNA was then sequenced by Sanger sequencing (Microsynth).

### Statistical Analysis

Statistical analyses were performed using Prism software (GraphPad). *Mann–Whitney Student’s t-test*, *Log-rank Mantel–Cox test* or *ANOVA* was used depending on the experiment, and groups were considered statistically significant if p < 0.05. In TC-1 tumor model, mice were stratified according to tumor size on the day of the first vaccination. In other experiments, mice were randomly assigned to the treatment on the day of the first vaccination.

### Ethic Approval

These studies have been reviewed and approved by the institutional and cantonal veterinary authorities in accordance with Swiss Federal law on animal protection.

## Results

### Combination of STING Agonist Treatment With a Protein Vaccine Modulates Peripheral CD8 and CD4 T Cell Response

**Figure 1:**
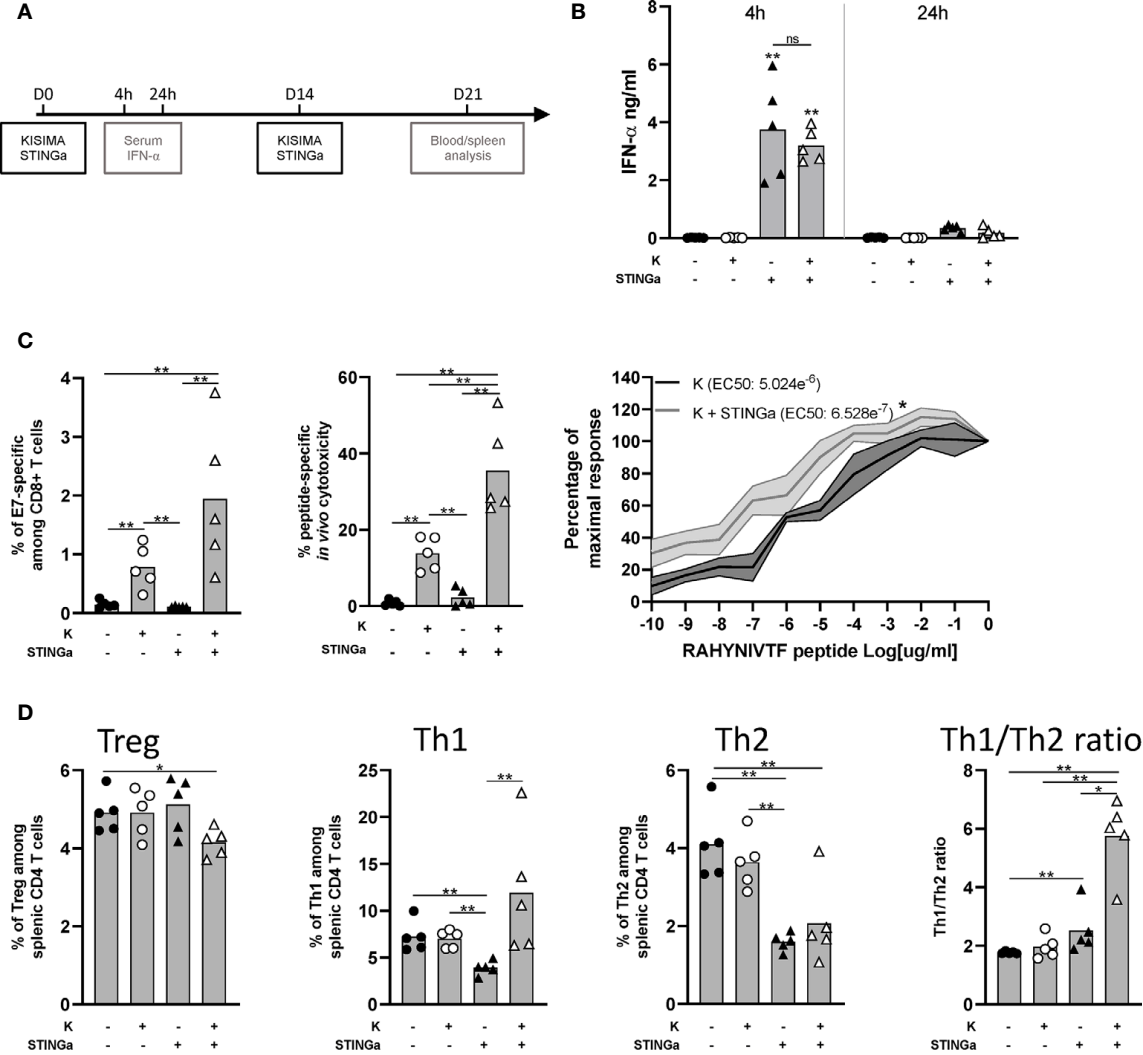
Protein vaccine combination treatment with STING agonist enhances functionality of CD8 T and CD4 T cell peripheral responses in tumor-free mice. C57BL/6 mice were treated with two administrations of Z13Mad25Anaxa vaccine, STING agonist or a combination of the two at two weeks interval. **(A)** Vaccination schedule. **(B)** Serum IFN-a level was measured 4 and 24 h post first vaccination. **(C)** One week after the second vaccination, circulating RAHYNIVTF (HPV-E7)-specific CD8 T cells were measured by multimer staining (left); *in vivo* cytotoxicity of RAHYNIVTF-specific CD8 T cells was measured by transfer of RAHYNIVTF peptide loaded splenocytes (middle); RAHYNIVTF-specific CD8 T cell TCR avidity was measured by *ex vivo* ELISpot (right). **(D)** Frequency of Treg (FoxP3^+^), Th1 (T-bet^+^), Th2 (GATA-3^+^) splenic CD4 T cells and Th1/Th2 ratio was measured by flow cytometry one week after the second vaccination. **(B–D)** One representative of two experiments is shown (n = 5/group/replicate), *Mann–Whitney test*, *p < 0.05, **p < 0.01; ns, not significant.

We previously reported that therapeutic subcutaneous (s.c.) vaccination with different KISIMA constructions elicits antigen-specific CD8 T cell response and promotes their infiltration within the tumor (19). In this study, therapeutic vaccination was combined with subcutaneous STING agonist (STINGa) administration. In preclinical tumor model and on-going clinical trials, STINGa is generally administered intra-tumorally (i.t.) in order to inflame the tumor microenvironment (TME). Subcutaneous STINGa injection in proximity of the vaccination site would allow for expanding its clinical application to non-accessible tumors while still exploiting the potent immune-stimulatory effect. In order to evaluate the impact of the combination on the T cells’ compartment, tumor-free mice were vaccinated twice at 2 weeks interval, with concomitant STINGa treatment (**Figure 1A**) and Z13Mad25Anaxa, a KISIMA-derived construct containing one human papilloma virus (HPV)-derived CD8 epitope (**Figure S1**). First, the systemic inflammatory response upon subcutaneous STINGa administration was analyzed. STINGa s.c. treatment induced a potent but transient systemic type I interferon response, characterized by high IFN-α serum level 4 h post-injection and already decreasing 24 h later (**Figure 1B**). The systemic interferon response was not affected by concomitant injection of the protein vaccine. Combination of Z13Mad25Anaxa and STINGa treatment further increased by two-fold the frequency of antigen-specific CD8 T cells (**Figure 1C**, left). In addition to their frequency, STINGa–Z13Mad25Anaxa combination treatment also highly enhanced the effector function of antigen-specific CD8 T cells. *In vivo* killing assay performed one week after vaccination revealed a significant 2.5-fold increase of antigen-specific cytotoxicity in STINGa–Z13Mad25Anaxa combination treated mice (**Figure 1C**, middle). Furthermore, *ex vivo* stimulation with decreasing concentration of HPV-CD8 peptide showed significantly higher TCR avidity on STINGa–Z13Mad25Anaxa primed T cells (**Figure 1C**, right). STINGa–protein vaccine combination modulated also bystander CD4 T cells response, deeply changing their polarization (**Figure 1D**). Significantly higher proportion of T helper 1 (Th1, T-bet^+^) and lower proportion of Treg (Foxp3^+^) and Th2 (GATA-3^+^) CD4 T cells were quantified in combination with STINGa, resulting in higher Th1/Th2 ratio. Similar modulation of CD8 and CD4 T cell response was observed using a different KISIMA construct containing CD4 and CD8 epitopes derived from ovalbumin (OVA), Z13Mad39Anaxa (**Figure S1**), suggesting that the modulation of the T cell response does not depend on the antigenic cargo (**Figure S2**). Z13Mad39Anaxa vaccination elicited polyfunctional CD8 and CD4 antigen-specific T cells, which produced IFN*γ* and TNFα following *ex vivo* stimulation with the specific peptide (**Figure S2**). Altogether, addition of STINGa to a protein vaccine profoundly impacts frequency and quality of CD8 T cell response along with polarization of CD4 T cell toward Th1.

### STINGa–Protein Vaccine Combination Inhibits B16-OVA Tumor Growth

**Figure 2:**
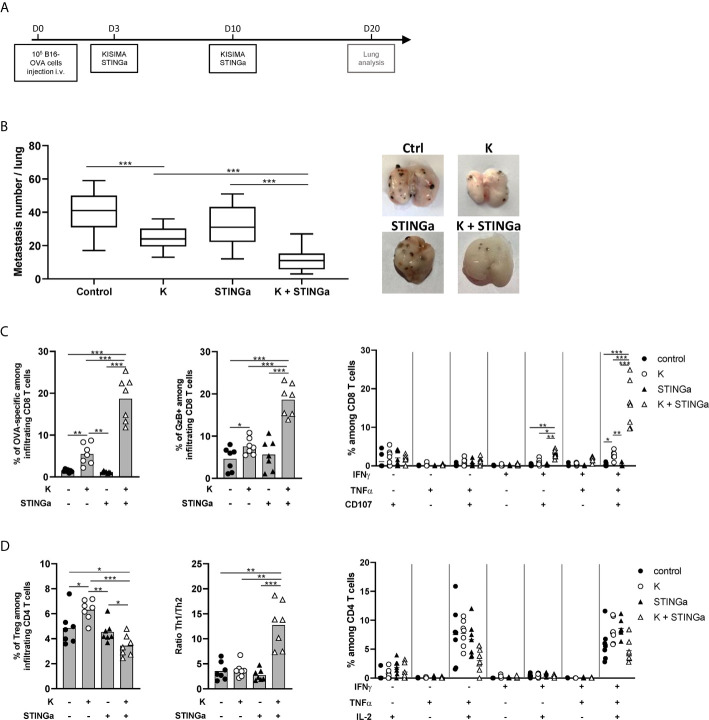
Combining protein vaccine with STING agonist inhibits B16-OVA tumor growth. 10^5^ B16-OVA cells were injected intravenously into C57BL/6 mice. At days 3 and 10 post tumor injection, mice were treated with two administrations of KISIMA vaccine, STING agonist or a combination of the two. At day 20, the number of lung metastasis was counted, and lung infiltrating lymphocytes were analyzed. **(A)** Vaccination schedule. **(B)** Number of metastatic nodules per lung and representative pictures. **(C)** Frequency of SIINFEKL (OVA)-specific CD8 T cells among tumor infiltrating leukocytes and expression of Granzyme B was measured by flow cytometry. Antigen-specific cytokine production by CD8 T cells was measured by intracellular staining after e*x vivo* stimulation with SIINFEKL peptide in presence of Golgi inhibitor. Antigen-specific cytokine production was measured by intracellular staining; frequency of cytokine-producing among CD8 T cells is shown. **(D)** Frequency of Treg (FoxP3^+^) and Th1/Th2 ratio was measured by flow cytometry. Antigen-specific cytokine production by CD4 T cells was measured by intracellular staining after e*x vivo* stimulation with ISQAVHAAHAEINEAGR (OVA-CD4) peptide in the presence of Golgi inhibitor. Antigen-specific cytokine production was measured by intracellular staining; frequency of cytokine-producing among CD4 T cells is shown. **(B–D)** One representative of two experiments is shown (n = 7/group/replicate), *Mann–Whitney test*, *p < 0.05, **p < 0.01, ***p < 0.001.

The anti-tumoral efficacy of therapeutic STINGa–protein vaccine combination treatment was then evaluated in the B16-OVA pulmonary metastases tumor model. Starting three days post tumor cell intravenous injection, mice were vaccinated twice at one-week interval, and the number of pulmonary metastasis was counted 10 days after the last vaccination (**Figure 2A**). Z13Mad39Anaxa vaccination resulted in a significant reduction of the number of metastasis, and while STINGa monotherapy had no effect, in combination with Z13Mad39Anaxa, it significantly further lowered the number of metastasis (**Figure 2B**). In addition, the presence and functionality of lung infiltrating lymphocytes (LILs) were analyzed by flow cytometry. The vaccination induced polyfunctional OVA-specific CD8 T cell infiltration, characterized by the expression of granzyme B (GzB), IFN*γ* and TNFα (**Figure 2C**), which were significantly increased with STINGa combination. Similar increase in T cell phenotype and functionality was observed in the periphery (blood and spleen) with a lower magnitude, suggesting that antigen-specific T cells are prevalently recruited to the tumor site (**Figures S3A, B**). As observed in tumor-free mice, KISIMA–STINGa combination treatment modulated the polarization of intra-tumoral CD4 T cells, decreasing the presence of Tregs while increasing the Th1/Th2 ratio (**Figure 2D**). *Ex vivo* stimulation with OVA peptide highlighted the presence of functional antigen-specific CD4 T cells in the spleen but not in the lungs, suggesting that helping CD8 T cell response is prevalently happening in the secondary lymphoid organ (**Figures 2D**, **S3C**).

Taken together these results show that combination treatment of a protein vaccine and a STINGa promotes both intra-tumoral infiltration of antigen-specific effector CD8 T cells and the functionality of peripheral CD4 T cells, resulting in the inhibition of B16-OVA tumor growth.

### Anti-Tumoral Effect of STINGa–Protein Vaccine Combination in TC-1 Tumor Model

**Figure 3:**
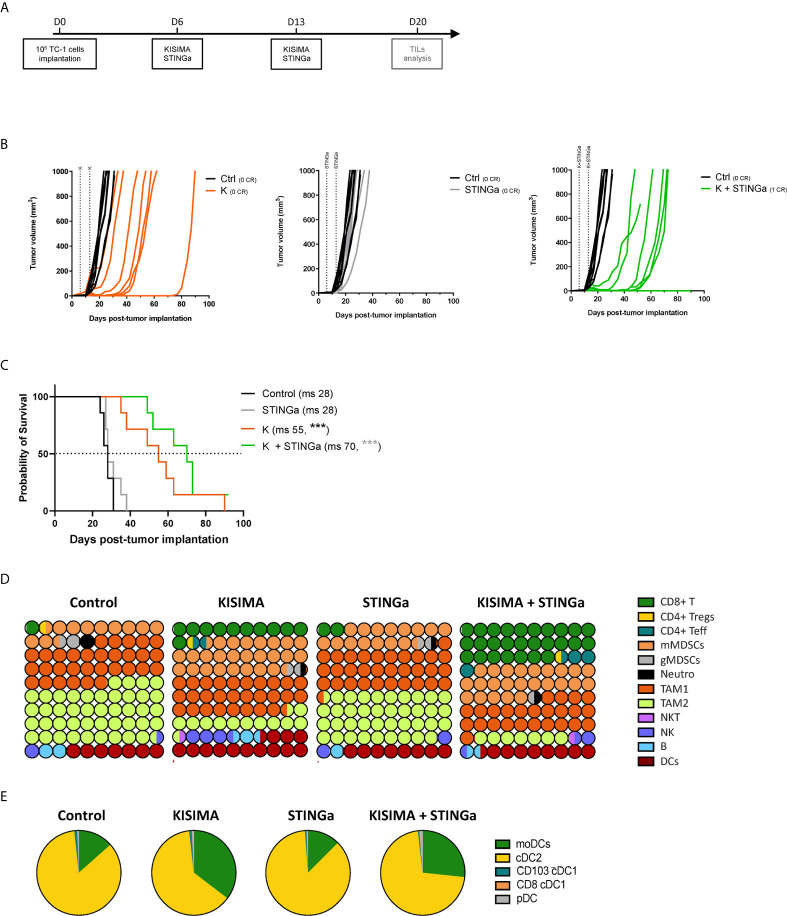
Combining protein vaccine with STING agonist delays TC-1 tumor growth and profoundly impacts tumor microenvironment. 10^5^ TC-1 cells were implanted subcutaneously on the back of C57BL/6 mice. When tumors were visible, mice were treated with two administrations of KISIMA vaccine, STING agonist or a combination of the two at one-week interval and tumor growth was monitored. **(A)** Vaccination schedule. Tumor growth **(B)** and median survival **(C)** were followed. CR, complete regression; ms, median survival. **(D)** 10 × 10 dot plot chart showing 100 circles, corresponding to 100%, and representing the proportion of different cell populations among CD45+ tumor-infiltrating cells; every circle represents 1% of the CD45+ population (see **Figure S11** for gating strategy). **(B)** Pie chart representing different tumor infiltrating dendritic cell populations, moDCs (CD11b^+^MHCII^hi^CD11c^hi^Ly6C^+^), cDC2 (CD11b^+^MHCII^hi^CD11c^hi^Ly6C^+^CD103^−^), cDC1 (CD11b-MHCII^hi^CD11c^hi^CD24^+^ and CD103^+^ or CD8^+^), pDC (CD11b^-^Ly6C^+^CD11c^int^B220^+^PDCA1^+^). One representative of three experiments (n = 7/group/replicate) **(B, C)** or a pool of two experiments (n = 7/group) **(D, E)** are shown. *Log-rank Mantel–Cox test*, ***p < 0.001.

The anti-tumoral effect of therapeutic STINGa–protein vaccine combination treatment was then assessed in TC-1 tumor—a cell line derived from mouse lung epithelial cells and transfected with HPV-16 E6/E7 and c-H-ras oncogenes. When tumors were palpable (day 6), mice were vaccinated twice at one-week interval, and tumor growth was monitored (**Figure 3A**). Z13Mad25Anaxa therapeutic vaccination of TC-1 tumor-bearing mice resulted in a significant delay of tumor development and a 27-day increase in median survival (**Figures 3B, C** **)**. While STINGa monotherapy had no effect on tumor growth, in combination with Z13Mad25Anaxa vaccination, it further delayed tumor development and enhanced median survival by 15 days compared to vaccination alone. Of note, neither single nor combination treatment caused significant variation of body temperature or weight shortly after administration to TC-1 tumor-bearing mice, indicating good safety and tolerability of the combination (**Figure S4**). Thus, therapeutic vaccination with a protein vaccine effectively delays TC-1 tumor growth, and concomitant STINGa treatment enhances the vaccine efficacy.

### Profound Impact of STINGa–Protein Vaccine Combination Treatment on the Composition of TC-1 Tumor Microenvironment

Despite T cells being the principal target of immunotherapy, due to their ability to directly kill cancer cells, the TME is a very complex network constituted by different immune cell types able to promote or inhibit cancer growth. Thus, the composition of TC-1 TME was dissected in order to have a complete view of its immunological status. TC-1 being a cold tumor model, CD4 and CD8 T cell infiltration combined represented less than 2% of tumor infiltrating CD45+ cells in vehicle treated mice (**Figures 3D**, **S5**). The most prominent cell type was tumor associated macrophages (TAMs), representing up to 75% of the infiltrate, and in particular the immunosuppressive TAM2. Myeloid derived suppressor cells (MDSCs) represent another 15%, with the monocytic type (mMDSC) being prevalent. Other cell types found with lower frequency were dendritic cells (DCs, 7%), B cells (2%), NK and NKT cells (1.5%), and neutrophils (1%). Therapeutic protein vaccine treatment induced a profound modification of the TME, characterized by a strong increase in CD8 T cells and DC frequency and the appearance of non-Treg CD4 T cells. Interestingly, the increase of DC infiltration was also characterized by an increase of monocytic DC (moDC) proportion (**Figures 3E**, **S6**), a particular subset which has been described to differentiate only in inflammatory conditions and has been shown to activate anti-tumoral T cell responses (21). While the TAM1 compartment remained mostly unaltered, TAM2 frequency was strongly decreased resulting in a higher TAM1/TAM2 ratio. In contrast, the frequency of mMDSC was increased by Z13Mad25Anaxa vaccination, while granulocytic MDSC and neutrophils remained mostly unchanged. STINGa monotherapy did not affect the composition of TME, which was essentially identical to vehicle treated mice. However, in combination with protein vaccine treatment, it further expanded both CD8 and non-Treg CD4 T cell infiltration by 2.5-fold, while decreasing TAM2 frequency.

In addition to TME cellular composition, the intra-tumoral expression of MHC-I and MHC-II was monitored. Both H2-Kb and H2-Db MHC-I allele expression was up-regulated by tumor cells in Z13Mad25Anaxa vaccinated or combination treated mice, compared to both vehicle and STINGa treatment (**Figure S7A**), suggesting that therapeutic protein vaccine treatment could even promote tumor cell recognition by CD8 T cells. At the same time, Z13Mad25Anaxa vaccination also increased MHC-II expression on CD11b+ cells (**Figure S7B**, right), thus promoting the presentation of epitopes to CD4 T cells.

Altogether, these results highlight the profound modulation of TME induced by therapeutic protein vaccine treatment, which is able to turn a cold tumor into hot tumor favoring the effect of STINGa treatment which further increases anti-tumoral immunity.

### Therapeutic STINGa–Protein Vaccine Combination Treatment Improves Antigen-Specific CD8 T Cell Response in TC-1 Tumor Bearing Mice

**Figure 4:**
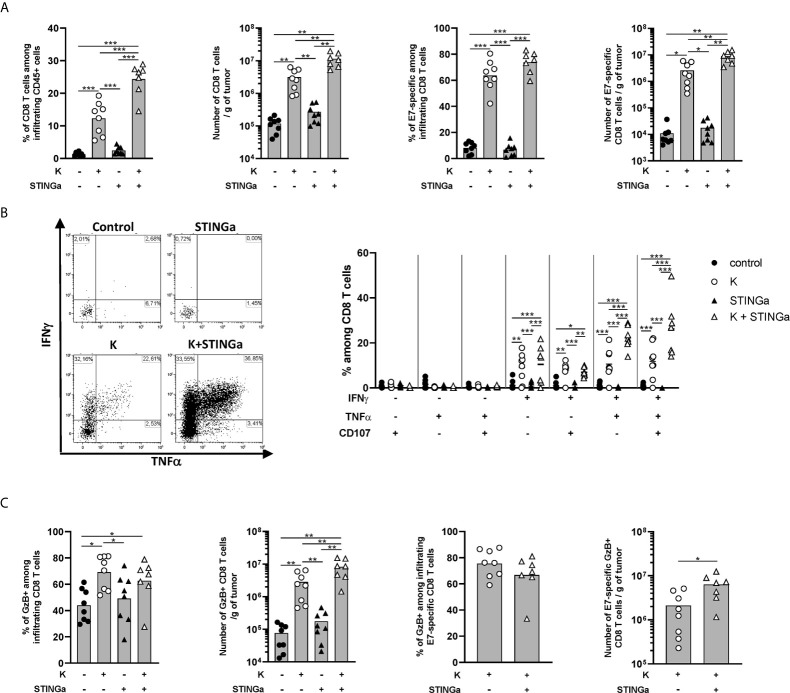
Combining protein vaccine treatment with STING agonist enhances functionality of intra-tumoral CD8 T cells in TC-1 model. 10^5^ TC-1 cells were implanted subcutaneously on the back of C57BL/6 mice. When tumors were visible, mice were treated with two administrations of KISIMA vaccine, STING agonist or a combination of the two at one-week interval. One week after the last treatment, mice were sacrificed, tumor harvested, and CD8 T cells’ presence and phenotype were analyzed by flow cytometry. **(A)** Frequency and number of total and RAHYNIVTF (E7)-specific CD8 T cells among tumor infiltrating leukocytes. **(B)** Tumor infiltrating CD45+ cells were stimulated *ex vivo* with RAHYNIVTF peptide in the presence of Golgi inhibitor. Antigen-specific cytokine production was measured by intracellular staining; representative FACS plots and frequency of cytokine-producing among CD8 T cells are shown. **(C)** CD45+ tumor infiltrating cells were cultured *ex vivo* with Golgi inhibitor and granzyme B production was monitored by intracellular staining. Frequency and total number of granzyme B-producing total and RAHYNIVTF-specific CD8 T cells are shown. **(A–C)** A pool of two experiments is shown (n ≥7/group), *Mann–Whitney test*, *p < 0.05, **p < 0.01, ***p < 0.001.

The effect of Z13Mad25Anaxa–STINGa combination on CD8 T cell response in TC-1 tumor-bearing mice was then analyzed. Protein vaccine treatment significantly increased peripheral HPV-specific response, and as expected, combination with STINGa further enhanced antigen-specific CD8 T cell number (**Figure S8A**). Very low levels of total or HPV-specific CD8 T cells were found in control mice, either considering proportion—they represented less than 1% of tumor infiltrating leukocytes—or total number (**Figure 4A**), a typical trait of cold tumors. Z13Mad25Anaxa vaccination induced a significant increase of CD8 T cell tumor infiltration, of which over 60% was HPV-specific. Notably, HPV-specific CD8 T cells were massively present within the tumor in contrast to the level observed in the blood, suggesting that measurement of peripheral responses can only partially predict the intra-tumoral outcome. While STINGa monotherapy did not modulate CD8 T cell tumor infiltration nor the proportion of HPV-specific, Z13Mad25Anaxa–STINGa combination significantly increased both CD8 T cell infiltration and HPV-specific proportion. In addition, the functionality of tumor-infiltrating HPV-specific CD8 T cells was monitored by measuring IFN*γ*, TNFα, and degranulating marker CD107*α* expression after HPV-specific *ex vivo* stimulation of TILs; a significant increase of HPV-specific cytokine-producing and degranulating CD8 T cells was found in Z13Mad25Anaxa-vaccinated mice compared to that in control or STINGa monotherapy group (**Figure 4B**). Combination with STINGa significantly further increased not only CD8 T cell functionality but also the frequency of multifunctional cells. Higher frequency and number of GzB-producing CD8 T cells in Z13Mad25Anaxa vaccinated mice were observed compared to vehicle or STINGa monotherapy (**Figure 4C**). Combination with STINGa did not impact the frequency of GzB-positive among HPV-specific CD8 T cells but further increased their total number (**Figure 4C**). In contrast to the intra-tumoral compartment, very low frequency of cytokine- or GzB-producing splenic HPV-specific CD8 T cells was observed in all the different treatments (**Figure S8B**).

Despite high activation, the majority of tumor infiltrating CD8 T cells in protein vaccine treated mice expressed PD-1, Tim-3, CD38, and NKG2a markers associated with T cell exhaustion (22, 23) (**Figures S9A–C**). Interestingly, in the combination group, a lower proportion of CD8 T cells co-expressed PD-1 and Tim-3, suggesting a less exhausted phenotype, which correlated with the higher proportion of cytokine-secreting cells. Similar to functionality analysis, peripheral CD8 T cells showed a less-exhausted phenotype (**Figure S8A** right), suggesting that exhaustion is acquired within the TME.

Taken together these results show that therapeutic protein vaccine treatment highly increases HPV-specific CD8 T cells tumor infiltration and functionality and while STINGa monotherapy has no effect, the combination further enhances vaccination efficacy.

### Therapeutic STINGa–Protein Vaccine Combination Treatment Modulates Intra-Tumoral CD4 T Cell Responses

**Figure 5:**
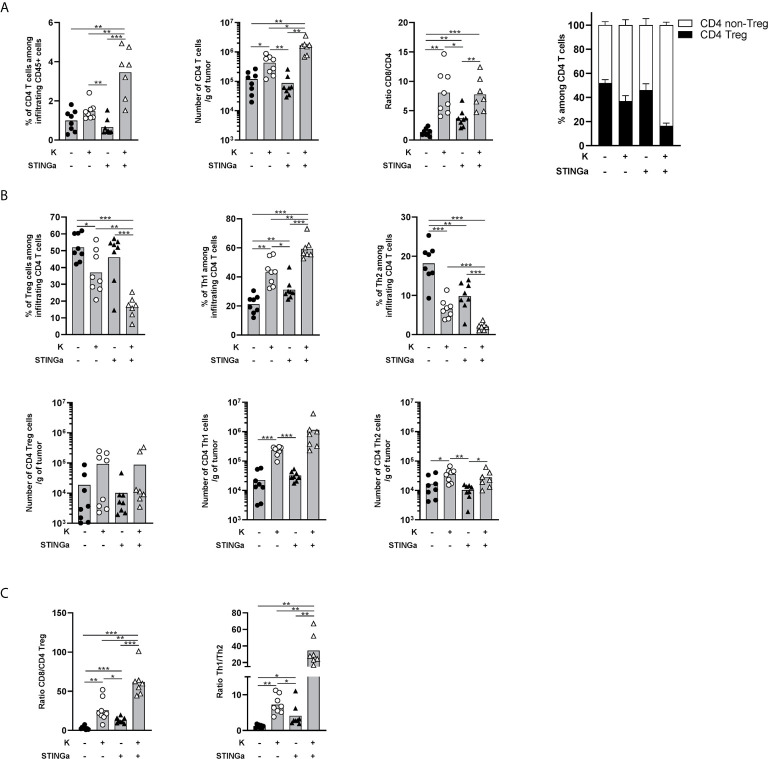
Combination of protein vaccine with STING agonist positively modulates the polarization of intra-tumoral CD4 T cells in TC-1 model. 10^5^ TC-1 cells were implanted on the back of C57BL/6 mice. When tumors were visible, mice were treated with two administrations of KISIMA vaccine, STING agonist, or a combination of the two at one-week interval. One week post the last treatment, mice were sacrificed, tumor harvested, and CD4 T cells’ presence and phenotype were analyzed by FACS staining. **(A)** Frequency and number of total CD4 T cells among tumor infiltrating leukocytes, ratio between tumor infiltrating CD8 T cells and CD4 T cells, frequency of Treg and non-Treg among tumor infiltrating CD4 T cells. **(B)** Frequency (top) and total number normalized to tumor weight (bottom) of Treg, Th1, and Th2 among tumor infiltrating CD4 T cells. **(C)** Ratio between tumor infiltrating CD8 T cells and CD4 Treg cells, and between Th1 and Th2 tumor infiltrating CD4 T cells. **(A–C)** A pool of two experiment is shown (n ≥7/group), *Mann–Whitney test*, *p < 0.05, **p < 0.01, ***p < 0.001.

The importance of CD4 T cells, in particular the Th1 subset, for the development of a proper anti-tumoral CD8 T cell response is now established (24, 25). Thus, intra-tumoral CD4 T cells were monitored and a significantly increased infiltration was observed in Z13Mad25Anaxa–STINGa combination treated mice compared to the other groups (**Figure 5A**). The ratio between intra-tumoral CD8 and CD4 T cells is often used as a predictive value for the immunological state of TME (26) and was found to be increased in protein vaccine or combination treated mice (**Figure 5A**). Interestingly, the increased CD4 T cell infiltration was led by effector rather than regulatory CD4 T cells (**Figure 5A**). Further analysis revealed that in combination treated mice, most of intra-tumoral CD4 T cells were Th1 (T-bet^+^), whose number significantly increased over 50-fold compared to that of the control group, while CD4 Tregs only slightly increased and just a minimal part was Th2 (GATA-3^+^) cells (**Figure 5B**). This modulation of bystander CD4 T cell polarization resulted in increased CD8/Treg and Th1/Th2 ratio, highlighting a less immunosuppressive TME (**Figure 5C**).

### TME Modulation and Epitope Mutation in Relapsing Tumors

**Figure 6:**
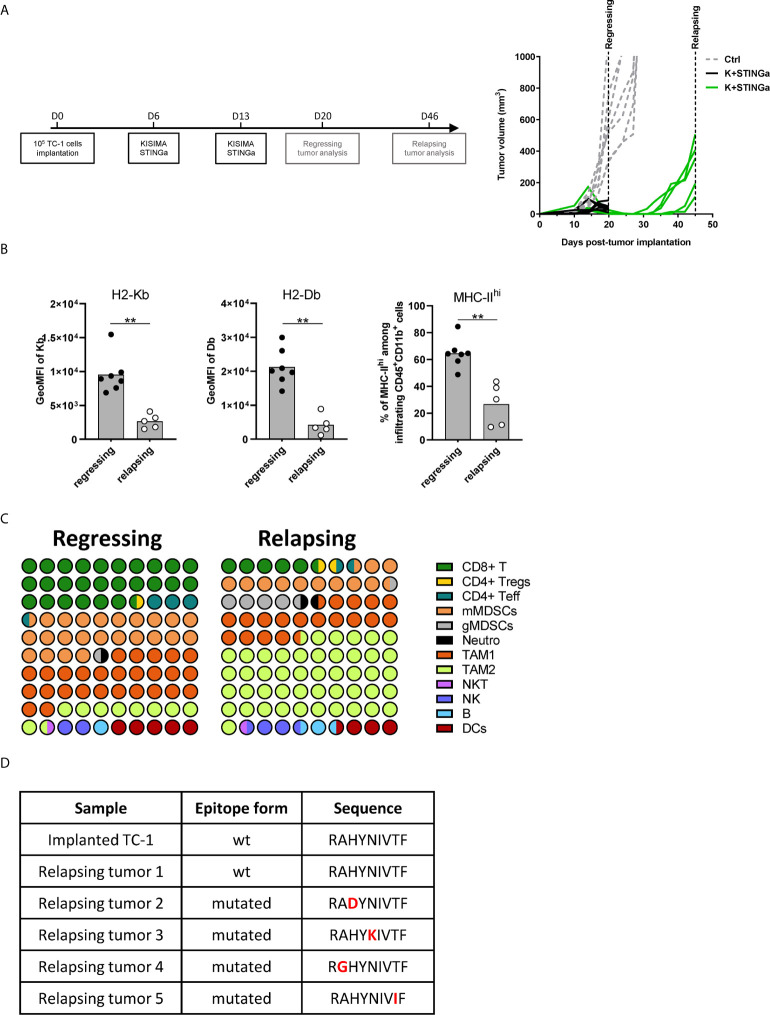
Comparison of regressing and relapsing tumor microenvironment. 10^5^ TC-1 cells were implanted on the back of C57BL/6 mice. When tumors were visible, mice were treated with two administrations of a combination of KISIMA vaccine and STING agonist at one-week interval. **(A)** Vaccination schedule and tumor growth. **(B)** Expression level of H2-Kb, H2-Db on CD45-tumor infiltrating cells and frequency of MHC-II^hi^ among CD11b+ cells. **(C)** One (regressing) or four (relapsing) tumors. One (regressing) or four (relapsing) weeks post the last treatment, mice were sacrificed, tumor harvested, and tumor microenvironment was analyzed by FACS staining. Proportion of different cell populations among CD45+ tumor-infiltrating cells is shown; every circle represents 1% of the CD45+ population (see **Figure S11** for gating strategy). **(D)** Sequence of HPV-E7 CD8 epitope expressed by implanted and relapsing tumors. **(B–D)** One representative of two experiments is shown (n ≥5/group/replicate), *Mann–Whitney test*, **p < 0.01.

Although STINGa–protein vaccine combination treatment was able to induce tumor regression in over 80% of mice and prolonged disease control, the majority (over 95%) of animals developed tumor relapses between two and four weeks after the last vaccination (**Figure 6A**). In order to understand the mechanism of tumor relapse, the expression of intra-tumoral MHC-I was measured, as its down-regulation by tumor cells is one of the tumor escape mechanisms (27). Indeed, MHC-I expression was down-regulated on relapsing tumor cells compared to that of regressing tumors (**Figure 6B**). In addition, the expression of MHC-II on CD11b+ cells was also down-regulated, suggesting that antigen-presentation to CD4 T cells was reduced (**Figure 6B**). To address the impact of decreased intra-tumoral antigen-presentation, the TME composition of escaping tumors was monitored. In contrast to regressing tumors, the TME was largely dominated by TAM-2, which represented over 45% of the total CD45+ infiltrates, followed by TAM-1 and mMDSC (**Figure 6C**), and resembled very closely to mock treated tumor (**Figure 3C**). CD8 T cells represented only the 5% of the immune infiltrate, a drastic reduction compared to the over 25% of regressing tumors. While the total number of antigen-specific CD8 T cells decreased by 10-fold in relapsing tumors (**Figure S10A**), their functionality was not impacted, with most of the cells still able to produce IFN*γ*, TNFα, and granzyme B following brief *ex vivo* peptide-specific stimulation (**Figure S10B**). The polarization of intra-tumoral CD4 T cells was impacted as well; the proportion of t-bet+ Th1 CD4 T cells remained unchanged; however, the frequency of anti-inflammatory Tregs and Th2 cells significantly increased, resulting in a less favorable Th1/Th2 ratio (**Figure S10C**).

Finally, as a prerogative of cancer cell is to be inclined to acquire new mutations, the HPV-16 E7 mRNA expressed by tumor cells, which contains the epitope encoded by Z13Mad25Anaxa vaccine, was sequenced. Surprisingly, in 80% of the mice the HPV-16 E7 transcript contained a single amino-acid mutation in the CD8 epitope region (RAHYNIVTF) (**Figure 6D**), which allowing tumor to escape recognition by Z13Mad25Anaxa elicited HPV-E7 specific CD8 T cells, and proliferate despite the presence of functional CD8 T cells. Taken together, these results highlight different tumor cell intrinsic and extrinsic immune evasion mechanisms which allow TC-1 tumor to finally escape from the protective tumor-specific response elicited by therapeutic STINGa–protein vaccine combination treatment.

## Discussion

The efficacy of KISIMA platform for development of protein based cancer vaccines which showed high immunogenicity and anti-tumoral efficacy in different preclinical tumor models was previously reported (19, 20). In preclinical studies, STING agonists have been mainly assessed using intra-tumoral injection, with the goal of directly inflame the tumor, which showed a potent anti-tumoral activity (13). The promising preclinical studies have been recently translated into the initiation of several clinical studies focusing on different tumor types, aiming to use STINGa as a universal cancer treatment. However, current reported clinical data do not corroborate the pre-clinical results (28).

In preclinical studies, STINGa anti-tumoral activity was shown to require intra-tumoral administration (29). Nevertheless, STINGa i.t. treatment induces also a systemic interferon response, which can result in abscopal efficacy on untreated tumors (29). This highlights the possibility of using STINGa in combination with a cancer vaccine, exploiting its potent immune-modulator properties in addition to the vaccine induced antigen-specific T cell response. In this combination setting, STINGa would not necessarily require i.t. administration, thus expanding its possible human indication to non-accessible tumors. We showed here that combination of KISIMA vaccination therapeutic protein vaccine with subcutaneous STINGa treatment profoundly impacts both quantity and quality of CD8 and CD4 T cells, which resulted in a prolonged control of tumor growth in both B16-OVA and TC-1 tumor models.

While treatment with a protein vaccine induced only a local inflammatory response, STINGa s.c. administration caused high level of systemic IFN-α, which impacted both CD8 and CD4 T cell responses. Combination with STINGa not only increased the frequency of splenic CD4 T cells, but also drove their polarization toward the inflammatory Th1 type and at the same time decreased Treg and Th2 frequency. Importantly, while the increase of total CD4 T cell frequency was strictly STINGa dependent, the different polarization required combination with protein-based vaccination, highlighting a combinatory effect on this cell type. CD4 T cell response has been widely overlooked in cancer immunotherapy, but recently gained more attention as Th1 and Th17 CD4 T cells have been shown to contribute to anti-tumoral immune responses by promoting CD8 T cell recruitment and activation or by secreting inflammatory cytokines (24, 25). It was recently reported that utilization of STINGa as adjuvant formulated within a *M. tuberculosis* protein subunit vaccine results in increased Th1 and Th17 *M. tuberculosis*-specific response (30); however to our knowledge this is the first report of a STINGa-dependent modulation of CD4 T cell polarization in a cancer immunotherapy context. In addition to the peripheral effects, the intra-tumoral T cell response was particularly increased after protein vaccine–STINGa treatment, highlighting the ability of the combination to promote tumor infiltration overcoming immune evasion and/or exclusion typical of TC-1 tumors. CD4 T cell frequency was highly increased upon combination treatment in TC-1 tumors, while it remained unchanged in STINGa monotherapy, highlighting again that vaccination is required for tumor infiltration in this tumor model. Intra-tumoral CD4 T cells have often been linked to immune-suppression due to their regulatory phenotype; however this was not the case in this combination, as Tregs represent only a minority of the infiltrating CD4 T cells, while the majority show a Th1 phenotype in both B16-OVA and TC-1 tumor models. However, following *ex vivo* stimulation with SIINFEKL peptide IFN-*γ*, TNF-α nor IL-2 production was increased in combination treated mice spleen but not tumor compartment, suggesting that antigen-specific CD4 T cells reside prevalently in secondary lymphoid organ. Nevertheless, the peripheral activity of antigen-specific CD4 T cells may be sufficient to help establish a more powerful CD8 T cell response.

In addition to CD4 T cells, therapeutic protein vaccine treatment highly enhanced CD8 T cell tumor infiltration and improved their TCR avidity and functionality—an effect further enhanced by combination with STINGa—while simultaneously increasing the expression of exhaustion markers PD-1 and Tim-3. Exhaustion being a multi-phased progressive process, intra-tumoral CD8 T cells could be in an early exhaustion phase and still maintain functionality, in particular as TILs were analyzed while tumor growth was controlled in vaccinated mice. Concordantly, antigen-specific CD8 T cells maintained their functionality weeks later in relapsing TC-1 tumors. An important difference was observed between the modest response induced by Z13Mad25Anaxa vaccination in peripheral blood and the magnitude of HPV-specific CD8 T cells observed within the tumor. This indicate that blood analysis is only partially representative of the anti-tumoral response induced by cancer vaccines, and its relevance in the prediction of vaccine immunogenicity in human patient should be carefully evaluated.

In addition to T lymphocytes, combination with STINGa induced profound changes of the TME, promoting the development of an inflammatory environment. The most evident modulation was the decrease of TAM2 frequency, which could be related to a lower tumor infiltration and/or to a different polarization of monocytes into mMDSCs, as their presence is increased by protein vaccine treatment. TAMs, in particular TAM2, have been associated with poor prognosis in several cancer types, promoting immune suppression, tumor growth and metastasis development (31). In preclinical models, TAM depletion or re-polarization towards the more inflammatory TAM1 type, was shown to favor tumor control and response to immunotherapy in different tumor models (32, 33). In addition to TAMs, Z13Mad25Anaxa vaccination also increased DC infiltration and their differentiation. The presence of intra-tumoral DCs is fundamental to maintain an active immune response, as they are able to pick up tumor antigens, migrate to the draining lymph node, and present them to T cells. Particularly important in cancer immune response are monocyte-derived (moDCs) cross-presenting DCs, which are able to activate tumor specific CD8 T cells and have been shown to play a primary role in the initiation of anti-tumoral immune responses (21). Protein vaccine treatment with or without STINGa combination increased the frequency of moDCs, suggesting that it can prolong the extent of the induction of immune response well past vaccination. Thus, therapeutic protein vaccine treatment in combination with STINGa bears additional beneficial effects to the induction of a potent antigen-specific CD8 T cells response.

Mechanistically, the impact of STINGa treatment in combination with a protein vaccine on T cell response is probably mediated by innate immune sensing, as direct STING activation in T cells was shown to induce cell death (34). In preclinical mouse studies, the anti-tumoral effect of STING signaling was closely associated with the potent induction of type I IFNs, which promoted the activation of cross-presenting Batf3-DCs resulting in increased CD8 T cells activation (35). In addition, activation of intra-tumoral Batf3-DCs was required for optimal trafficking of CD8 T cells into the core of the tumor, a process mediated by CXCL9 secretion (36). In the present study, STINGa monotherapy did not expand intra-tumoral DCs nor improved CD8 T cell infiltration, likely due to the distal administration route. Nevertheless, in combination with KISIMA vaccination, STINGa strongly enhanced CD8 T cell response, suggesting that a similar improvement of cross-presentation could take place at the vaccine draining lymph node. In addition, the STINGa-dependent polarization of CD4 T cells into Th1 is likely driven by the strong type I IFN response, which was also shown to impact CD4 T cell polarization (37).

While protein vaccine–STINGa combination treatment was able to control TC-1 tumor early growth and induce tumor regression, in the majority of the case, tumors were finally able to escape immune surveillance. TC-1 tumor escape following therapeutic vaccination was previously observed and was associated with a decreased tumor infiltration by inflammatory myeloid cells (38). Moreover, tumor regrowth was observed despite the presence of functional antigen-specific CD8 T cells. Similarly, in this study tumor relapses were associated with increased infiltration of immunosuppressive TAM-2 and MDSCs and a decrease of TAM-1, while CD8 T cells maintained their functionality despite the reduction in number. However, in addition, tumor relapse was associated with single amino-acid mutations in the HPV-16 E7 CD8 epitope contained in Z13Mad25Anaxa vaccine, highlighting the importance of including different antigen targets in human vaccine candidates. In this study, protein vaccine–STINGa combinatory treatment was administered only twice; however, the observation of mutations in the targeted epitope suggests that additional vaccinations using the same vaccine construction would not prevent tumor escape. Importantly, tumor escape associated with a single epitope mutation suggests that, despite the profound modulation of the tumor microenvironment induced by protein vaccine–STINGa combinatorial treatment, epitope spreading might be limited. The mutation within the epitope region differed from tumor to tumor, suggesting that it is the result of a random mutation rather than a driver mutation. Further studies are required to expand the sequencing of E7 antigen in relapsing tumor to a larger sample size allowing identification of the most recurrent mutations to be included in a new vaccine strategy which could potentially prevent tumor escape. In addition, in relapsing tumors, the expression of both MHC-I on tumor cells and MHC-II on CD11b+ cells were reduced, highlighting a decreased antigen presentation.

In conclusion, it is nowadays clear that an effective cancer immunotherapy cannot focus on a single treatment but must combine different approaches to target different aspects of tumor biology (39). Our findings highlight the promising combination of protein-based cancer vaccine with STING agonists and could offer opportunity for bimodal treatment of patients with innate resistance to immune check point blockade.

## Data Availability Statement

The raw data supporting the conclusions of this article will be made available by the authors, without undue reservation.

## Ethics Statement

The animal study was reviewed and approved by the institutional and cantonal veterinary authorities in accordance with Swiss Federal law on animal protection.

## Author Contributions

MD and EB conceived and designed the study. MR, SC, WB-B, and ER performed, processed, and analyzed *in vivo* mouse tumor studies and data. MD, EB, MR, and M-LS-R contributed to discussions and writing of the manuscript. All authors contributed to the article and approved the submitted version.

## Conflict of Interest

All authors were employed by company Boehringer Ingelheim International GmbH and AMAL Therapeutics.
