# Supplementary figures and images for: STING Agonist Combined to a Protein-Based Cancer Vaccine Potentiates Peripheral and Intra-Tumoral T Cell Immunity
Source: Front Immunol. 2021 Jul 1;12:695056. doi: 10.3389/fimmu.2021.695056 (PMC8283310; doi:10.3389/fimmu.2021.695056)

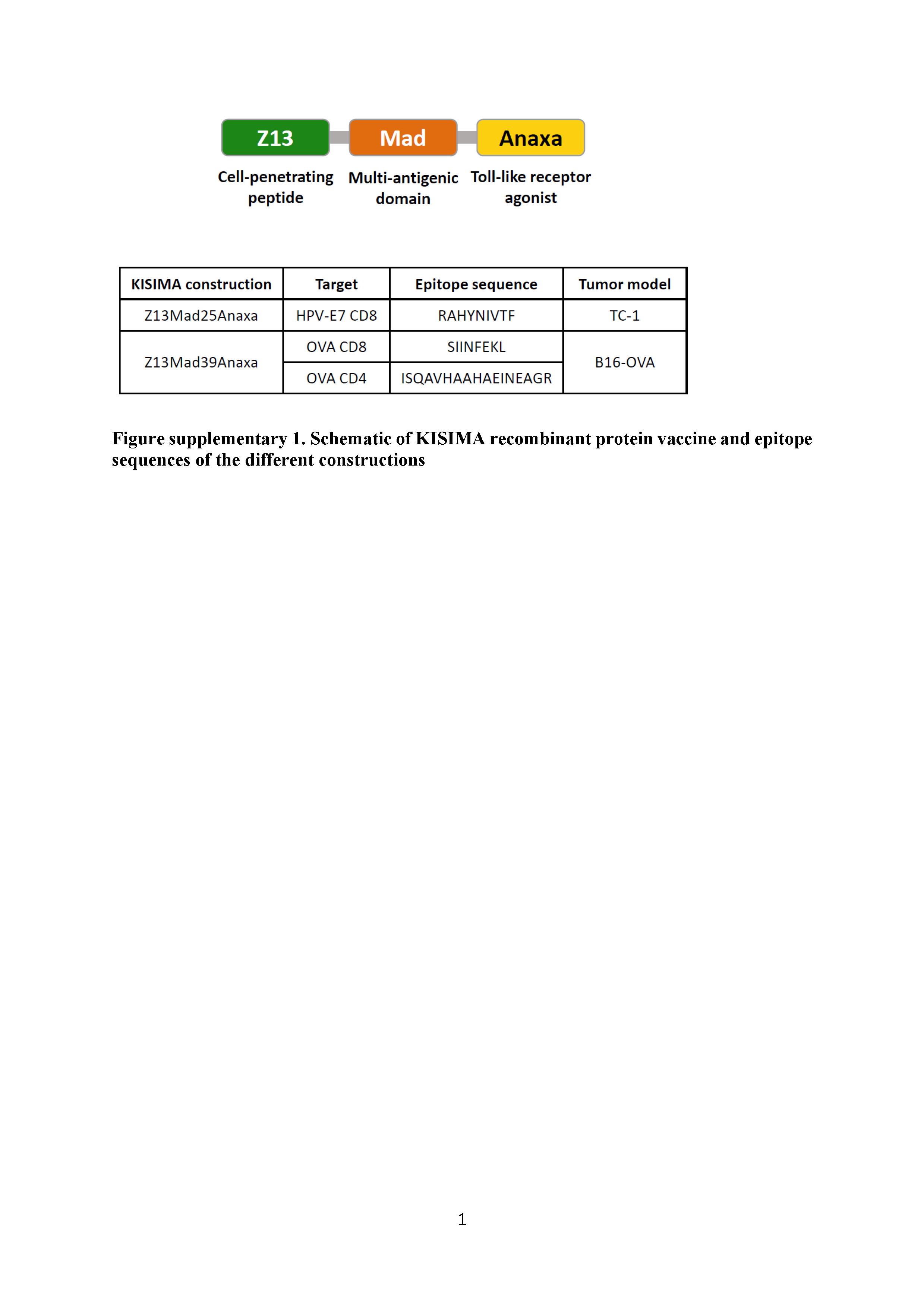

Supplement: Supplementary file 1 [file Image_1.tiff]

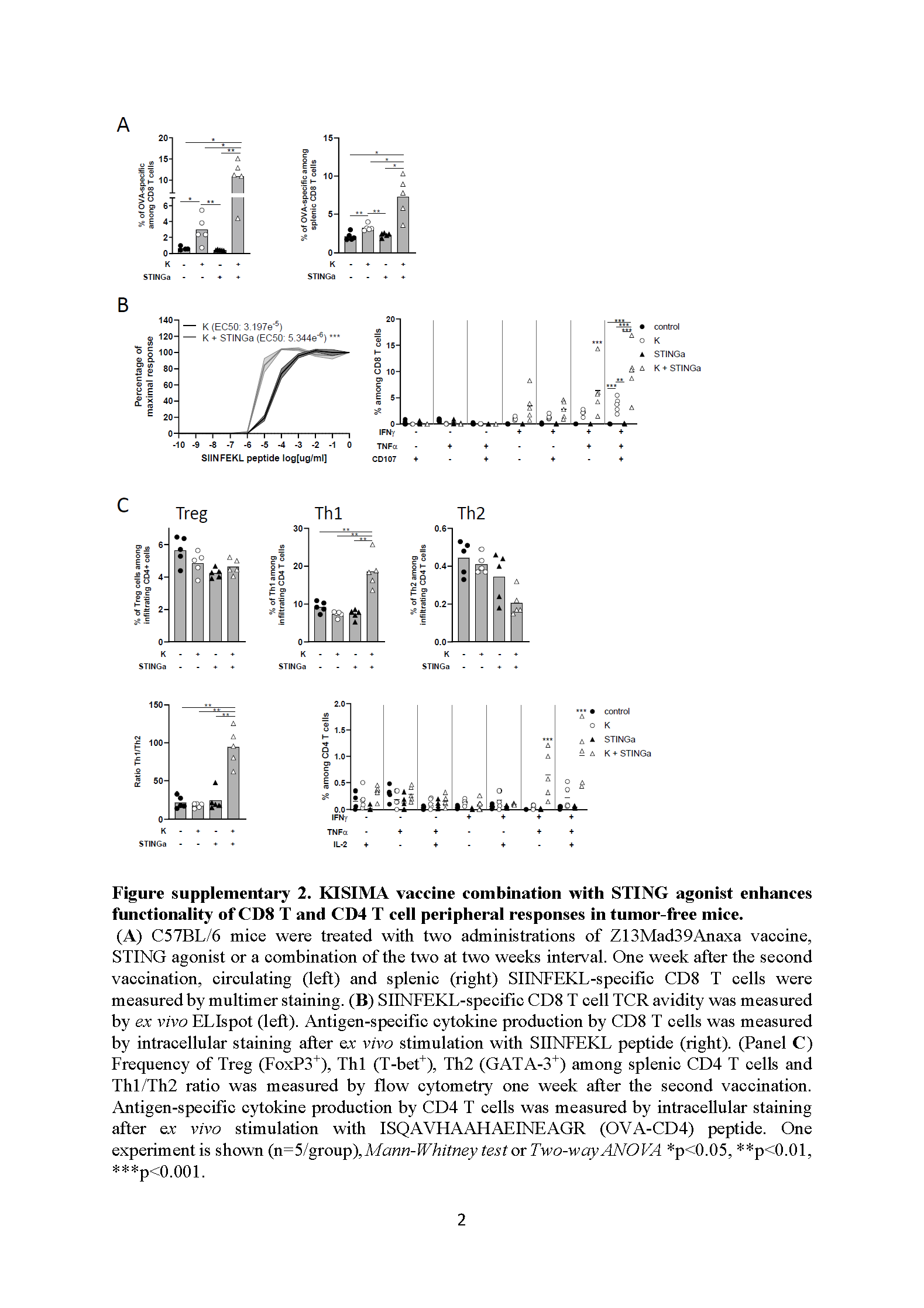

Supplement: Supplementary file 2 [file Image_2.tiff]

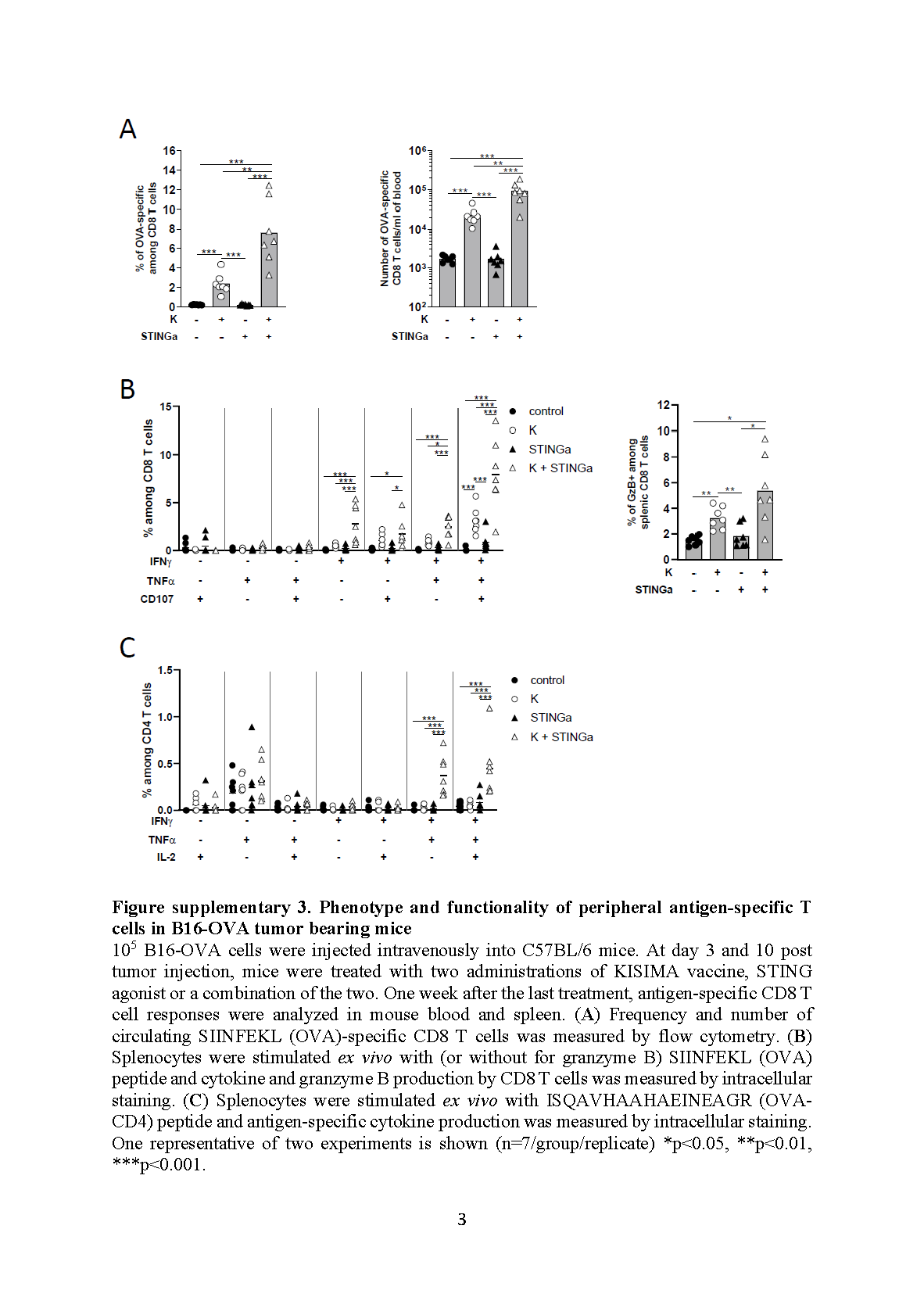

Supplement: Supplementary file 3 [file Image_3.tiff]

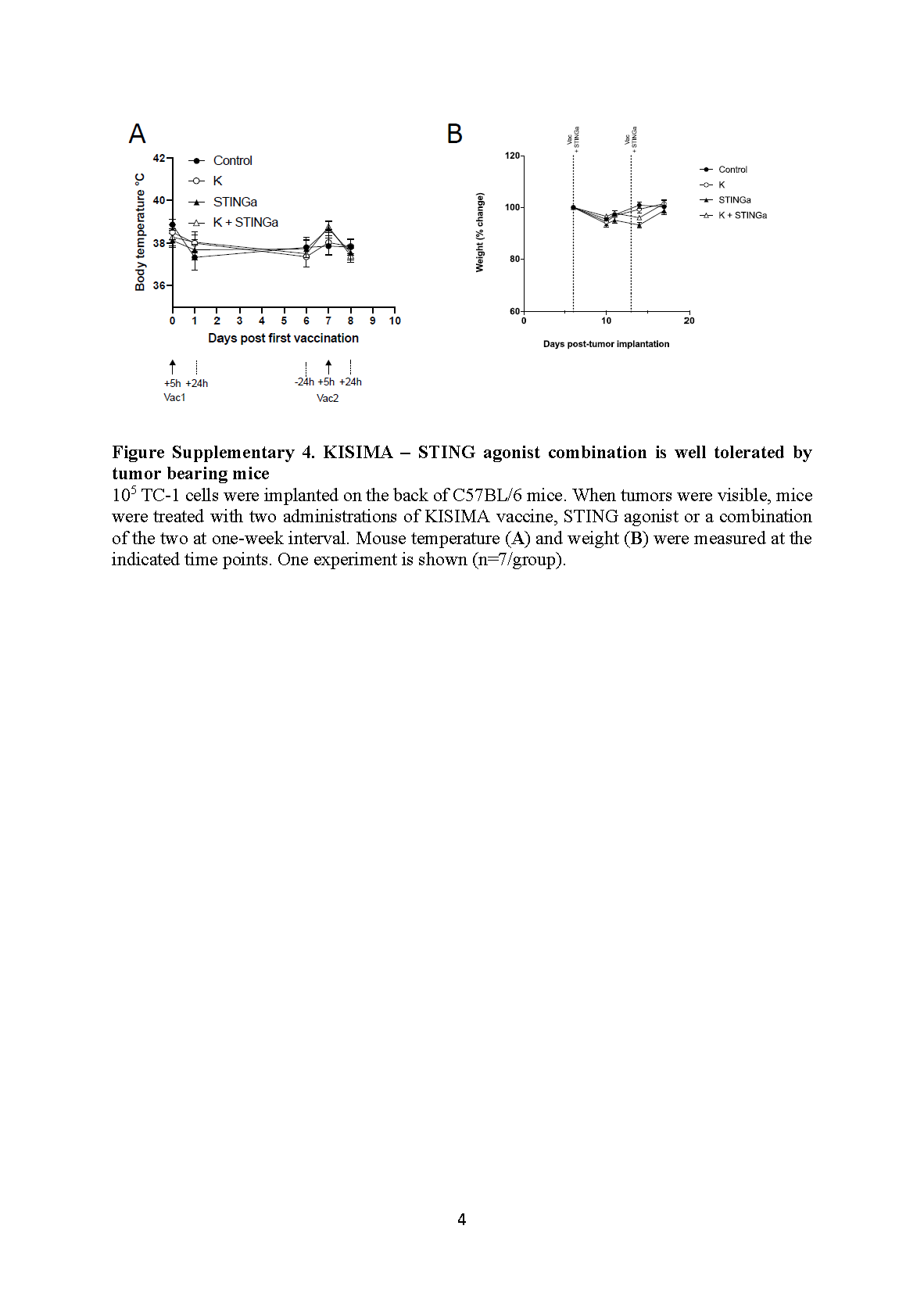

Supplement: Supplementary file 4 [file Image_4.tiff]

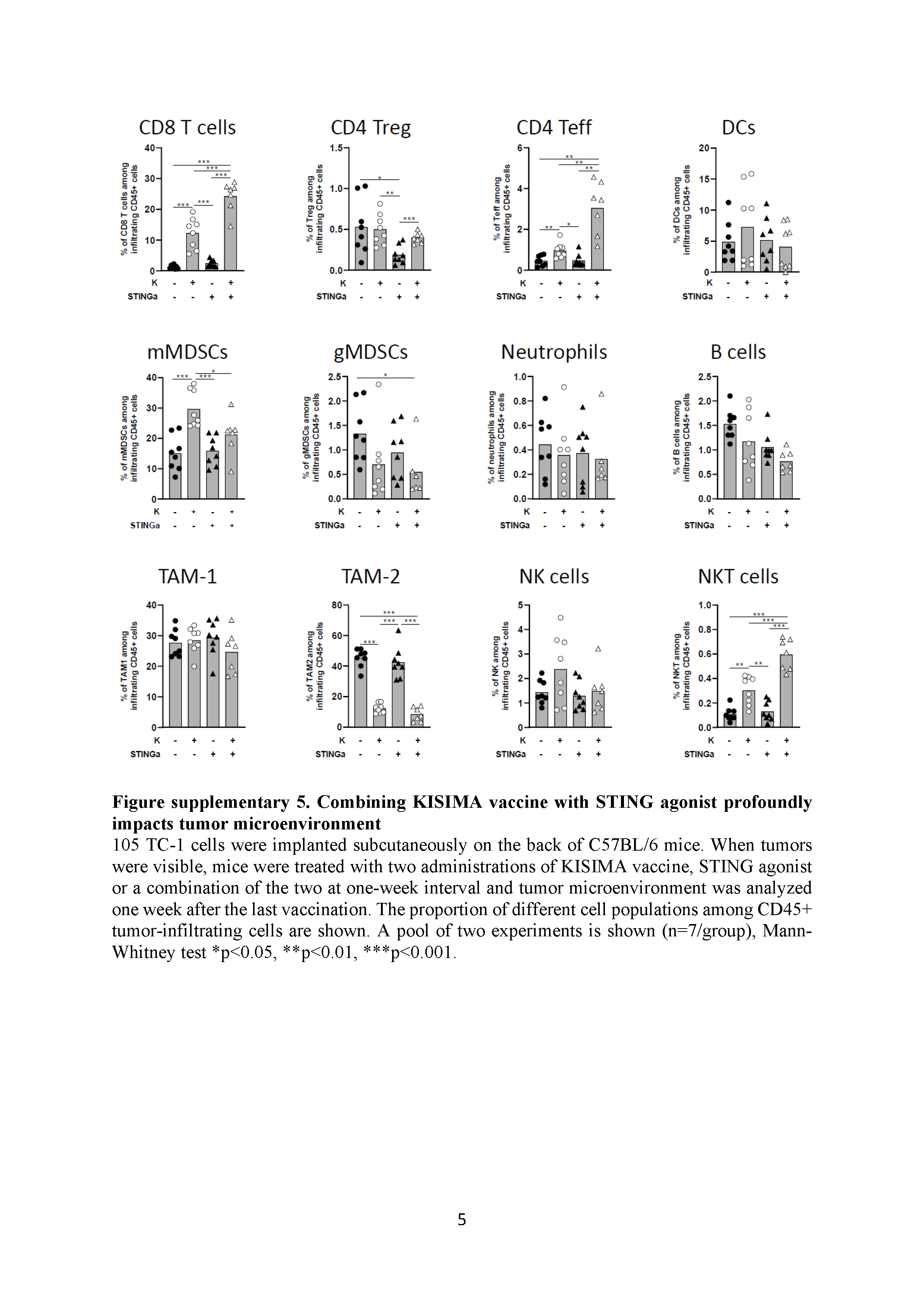

Supplement: Supplementary file 5 [file Image_5.tiff]

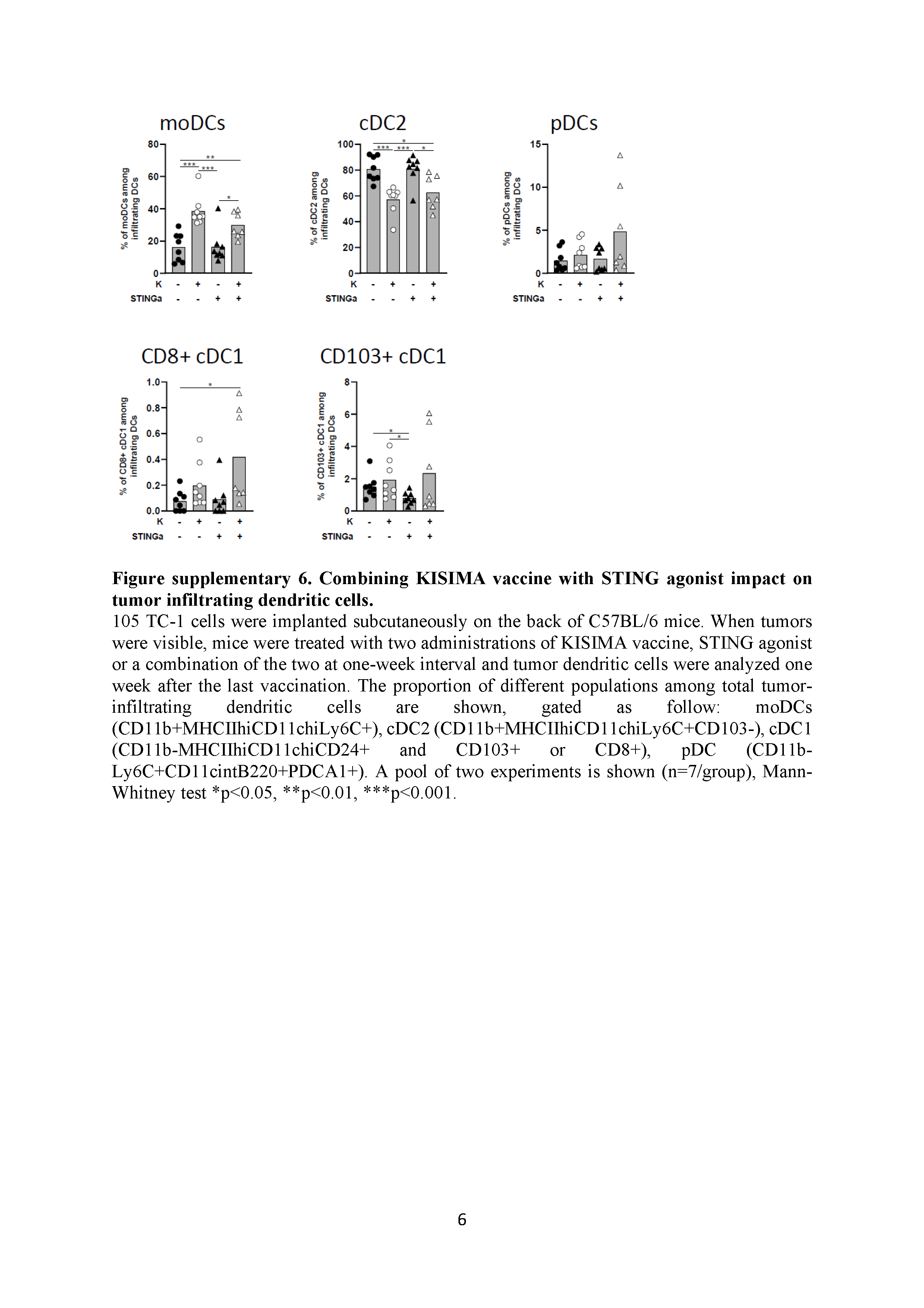

Supplement: Supplementary file 6 [file Image_6.tiff]

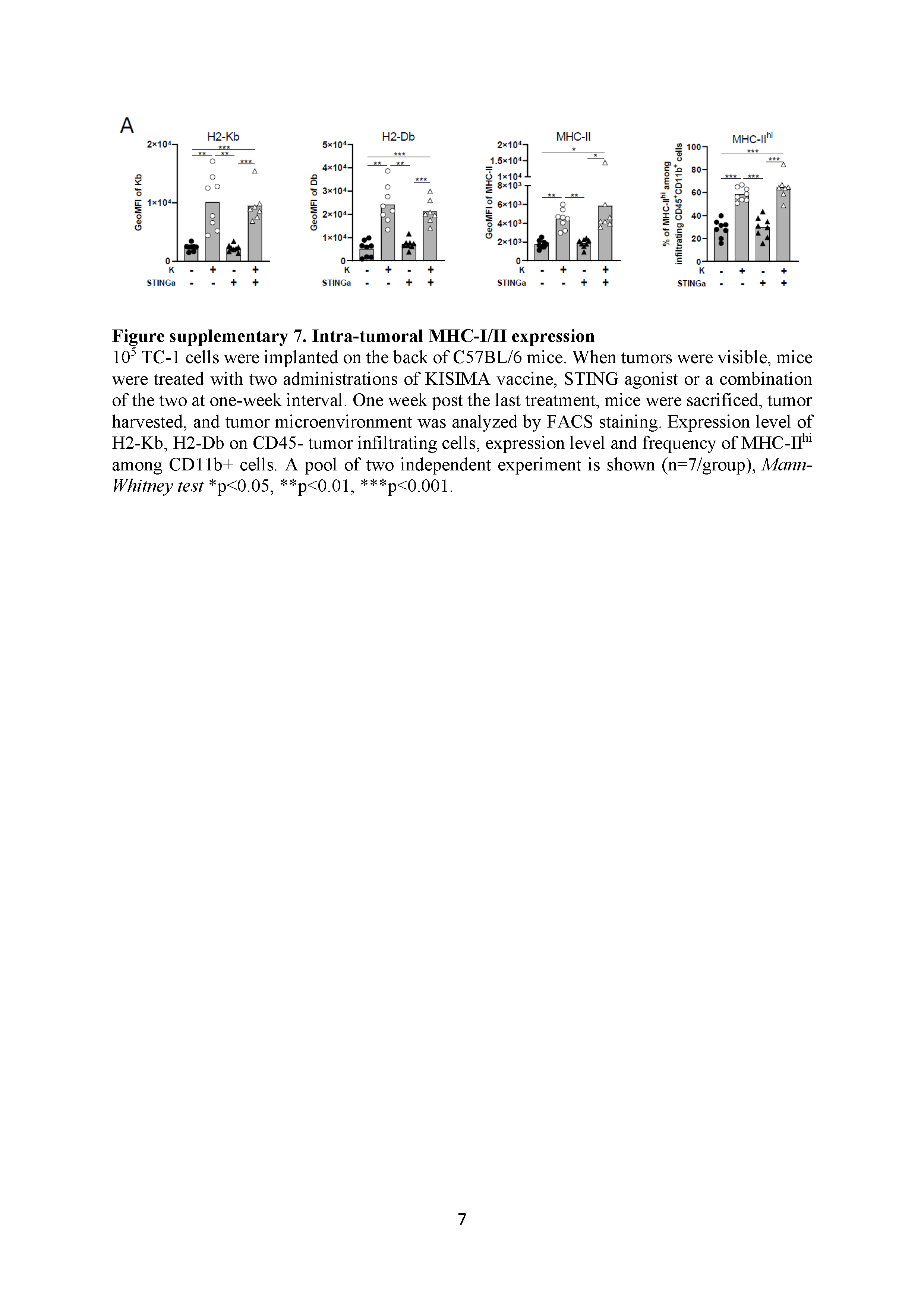

Supplement: Supplementary file 7 [file Image_7.tiff]

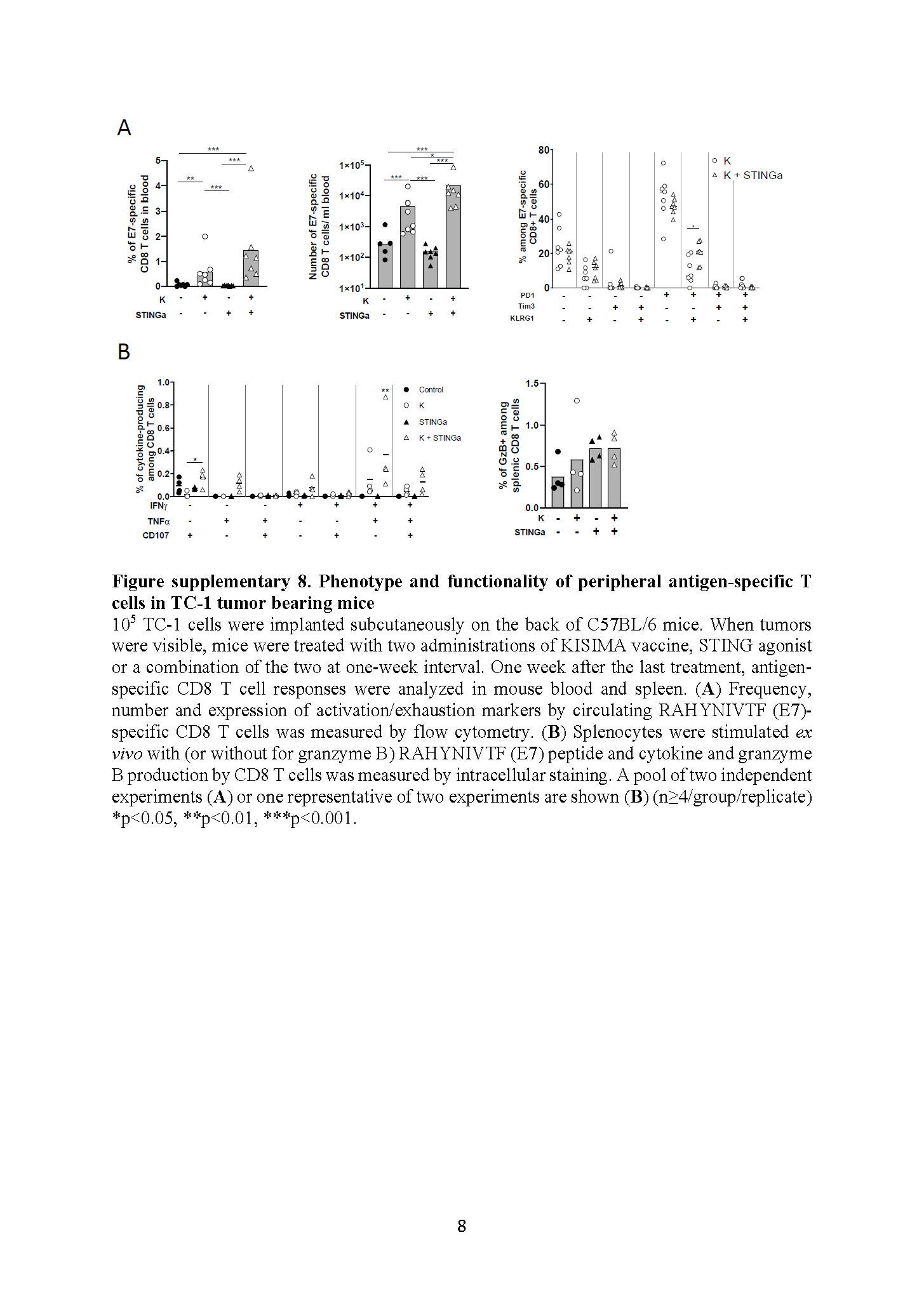

Supplement: Supplementary file 8 [file Image_8.tiff]

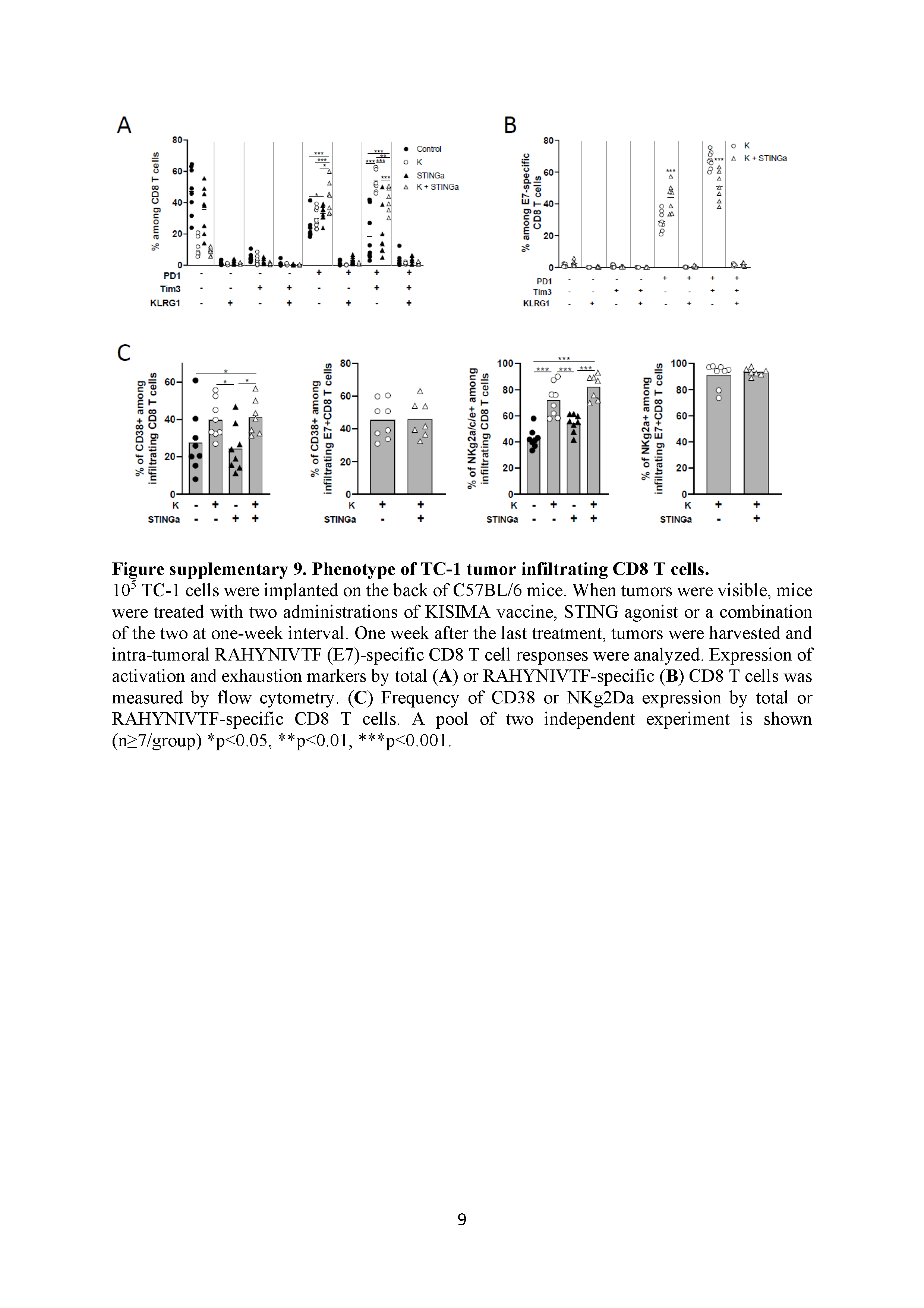

Supplement: Supplementary file 9 [file Image_9.tiff]

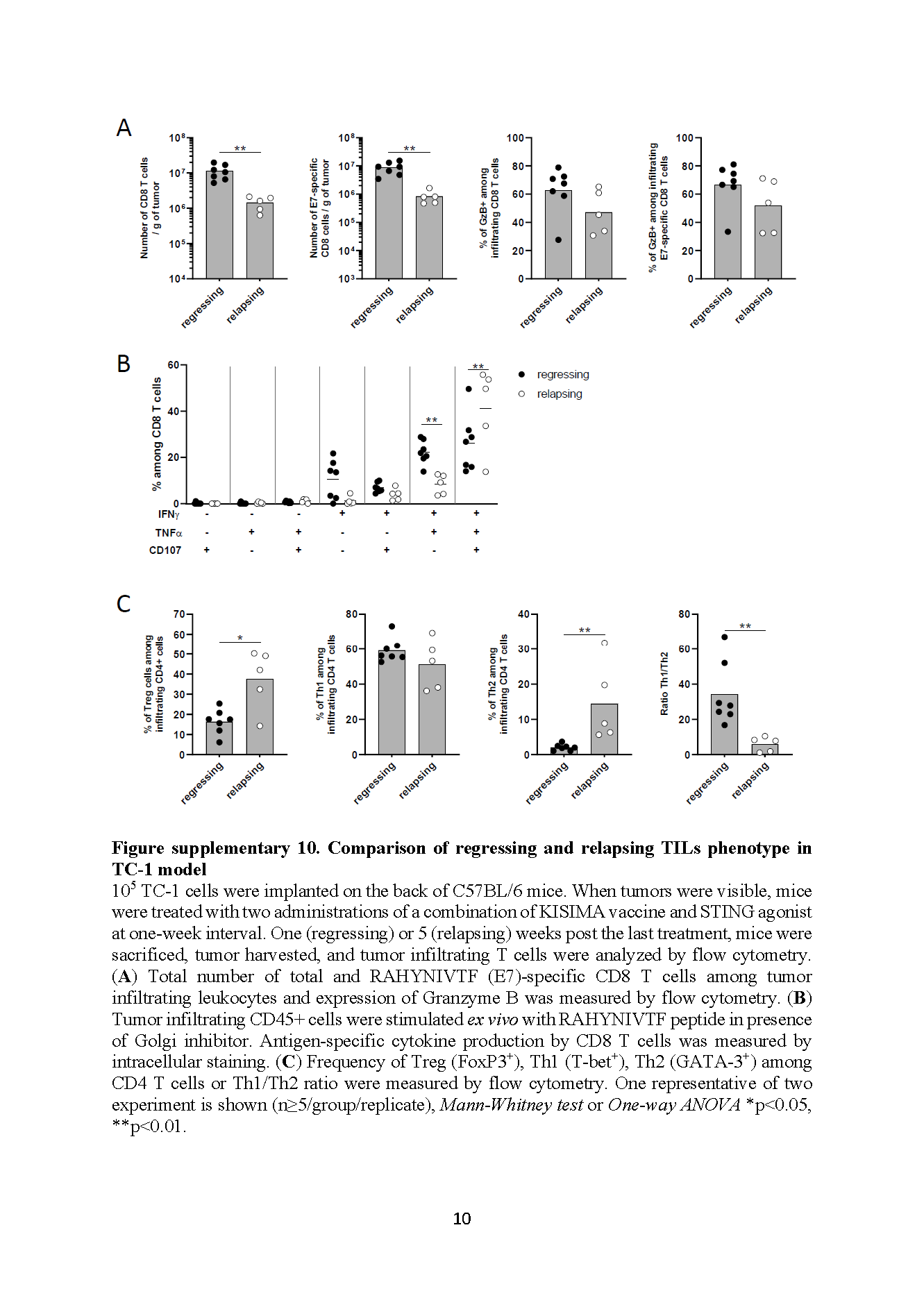

Supplement: Supplementary file 10 [file Image_10.tiff]

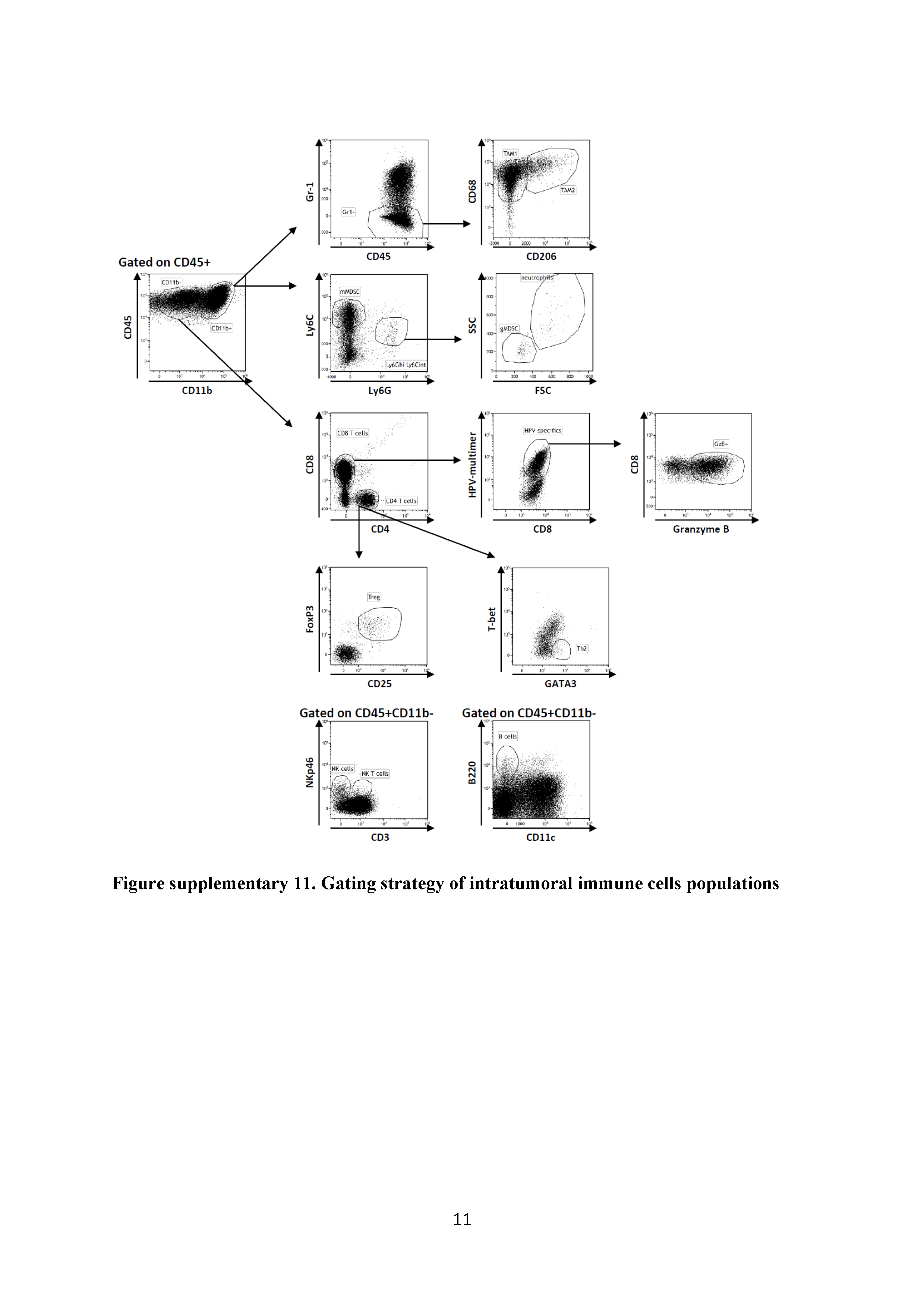

Supplement: Supplementary file 11 [file Image_11.tiff]
